# Bacterial Contamination on Latrine Surfaces in Community and Household Latrines in Kathmandu, Nepal

**DOI:** 10.3390/ijerph16020257

**Published:** 2019-01-17

**Authors:** Shannon McGinnis, Dianna Marini, Prakash Amatya, Heather M. Murphy

**Affiliations:** 1 Water, Health, and Applied Microbiology Lab (WHAM Lab), College of Public Health, Temple University, Philadelphia, PA 19122, USA; smcginnis@temple.edu; 2 Aerosan Toilets, Halifax, Nova Scotia B4A 4J8, Canada; dianna@aerosantoilets.ca (D.M.); prakash@aerosantoilets.ca (P.A.)

**Keywords:** WASH, community sanitation, global health

## Abstract

A lack of sanitation infrastructure is a major contributor to the global burden of diarrheal disease, particularly in low-income countries. Access to basic sanitation was identified as part of the 2015 United Nations Sustainable Development Goals. However, current definitions of “basic” sanitation infrastructure exclude community or shared sanitation, due to concerns around safety, equity, and cleanliness. The purpose of this study was to measure and compare bacterial contamination on community and household latrine surfaces in Kathmandu, Nepal. One hundred and nineteen swab samples were collected from two community and five household latrines sites. Community latrine samples were taken before and after daily cleaning, while household samples were collected at midday, to reflect normal conditions. Concentrations of total coliforms and *Escherichia coli* were measured using membrane filtration methods. Results found almost no differences between bacterial contamination on latrine surfaces in community and household latrines, with the exception of latrine slabs/seats that were more contaminated in the community latrines under dirty conditions. The study also identified surfaces with higher levels of contamination. Findings demonstrated that well-maintained community latrines may be as clean, or cleaner, than household latrines and support the use of community latrines for improving access to sanitation infrastructure in a low-income country setting.

## 1. Introduction

As of 2017, an estimated 2.3 billion people lacked access to improved sanitation facilities, worldwide [1]. Inadequate access to sanitation and hygiene facilities is known to be a leading cause of morbidity and mortality, particularly in low-income countries [2,3]. In fact, approximately 10% of the global burden of disease is thought to be attributed to inadequate water, sanitation, and hygiene (WASH), which is largely driven by increased exposure to human pathogens transmitted via the fecal-oral route [3]. A lack of safely managed WASH infrastructure may drive exposure to enteric pathogens through ingestion of water that is contaminated by improper waste disposal from latrines, improper hand hygiene, or through direct contact with contaminated latrine surfaces [1,4,5].

Due to the large health burden associated with inadequate sanitation and hygiene, improving access to safe sanitation and hygiene facilities has been identified as the key goal in the 2000 Millennium Development Goals, as well as the 2015 Sustainable Development Goals [6]. To ensure that adequate needs are met, the World Health Organization and United Nations International Children’s Emergency Fund (WHO/UNICEF) Joint Monitoring Programme for Water Supply, Sanitation, and Hygiene (JMP) set to monitor progress towards ensuring global access to “basic” level sanitation services. Importantly, these “basic” services, by definition, only include private facilities, which are not shared with other households [1]. This definition leaves shared or community sanitation facilities out of the “basic” sanitation requirements, due to concerns around accessibility, safety, gender equity, and cleanliness [7,8]. However, among the 2.3 billion people, worldwide, who lack access to basic sanitation, approximately 600 million rely on improved sanitation facilities that are shared with other households [1]. 

While some studies have identified health risks associated with using a shared sanitation facility (rather than a private facility), there is still an overall lack of evidence to support that there is a significant difference in cleanliness and subsequent health benefits between private and shared sanitation facilities [8,9]. In addition, the JMP’s 2017 report notes that while private toilets may remain the ultimate goal of improved sanitation programs, high-quality shared sanitation facilities may be the best option in some low-income urban settings [1]. Further, an increase in shared sanitation facilities may provide communities with the opportunity to move away from open defecation and begin to move up the sanitation ladder [7]. 

Due to the large number of individuals around the world relying on shared sanitation facilities and the important role these facilities play in growing urban centers, it is important for more research to be done to compare the cleanliness of these shared sanitation or community facilities with private or household latrines. Additionally, research is needed to identify community latrine surfaces at higher risk for disease transmission, to advise future cleaning interventions. For this reason, the purpose of this research was to quantify bacterial contamination on latrine surfaces in community and household latrines in Kathmandu, Nepal, to compare bacterial contamination at these sites and to identify surfaces that may be more likely to contribute to disease transmission.

## 2. Materials and Methods

### 2.1. Site Selection 

Kathmandu is the capital city of Nepal, a country that is currently listed as a least-developed country by the United Nations [10]. Although only 17 percent of Nepal’s population currently live in urban areas, Nepal is one of the top-ten fastest urbanizing countries in the world, with the population of Kathmandu expecting to double within the next, approximately, 50 years (from 2011 population estimates) [11,12]. Due, in part, to this rapid population growth and urbanization, as well as Nepal’s lack of financial resources and impacts of the 2015 earthquake, Kathmandu is currently facing a significant shortage of sanitation facilities [13]. In fact, the most recently published Nepali Household Survey (2015/2016) found that 19% of all households in Nepal did not have access to safe toilets, and among the poorest residents, this percentage is as high as 44% [14]. For this reason, both household and community sanitation facilities are important for improving access to much needed sanitation facilities in Kathmandu.

Two community toilet sites and five households were selected for swabbing in Kathmandu. Community toilet sites were privately-owned, pay-per-use toilets, with relatively high usage of about 1700 users per day (unpublished survey data). Both community sites were nearby the Thamel neighborhood of Kathmandu and had separate male and female toilet blocks, containing 2–4 pit latrines with slabs. In addition, both community toilet sites had, at least, one handwashing station with soap and water provided.

Household sites were selected to include a variety of different household types and income levels. These included three “Western-style” or cistern flush toilets located in one multi-family and two single-family households, as well as two pit-latrines (with slabs), located at one legal slum settlement and one illegal slum settlement, in Kathmandu (both multi-family private households). These sites were located near the community toilet sites, to assess a similar population to community latrine users. The three households with “Western-style” toilets had handwashing stations with soap on site, while the two slum settlements did not.

In addition to samples collected in Kathmandu, samples were also collected from a university toilet site located at Temple University in Philadelphia, Pennsylvania, USA in April 2018, as a control site. The toilet site at Temple University was selected as one of the most frequently used toilets at the university and consisted of separate male and female toilets with 2–3 “Western-style” or cistern flush toilets, as well as 2–3 handwashing stations with soap and water. Given that community sanitation in many parts of the developed world are considered an acceptable and hygienic form of sanitation, the site was used for comparison purposes.

### 2.2. Community Toilet Site Swabbing

At both Kathmandu community toilet sites and the university toilet control site, samples were collected from two female and three male cabins and from all available handwashing stations (this covered 100% of the female cabins and 60% of the male cabins at Site 1, 50% of the female cabins and 100% of the male cabins at Site 2, and 50% of the female and 100% of the male cabins at the control site). At the community toilet sites in Kathmandu, swabs were taken from the same surfaces in the same cabins, during both “dirty” and “clean” conditions. As cleaning occurred once per day at the end of the day, dirty samples were taken at the end of the day (around 5/6pm, prior to cleaning) and clean samples were taken early in the morning, after cleaning and prior to the toilets opening to the public. At the university control site, the samples were collected during the “dirty” conditions, (at approximately 5 pm, following a full day of use).

### 2.3. Household Toilet Swabbing 

Unlike the community toilet sites, household samples were collected around mid-day and “clean” or “dirty” conditions were not accounted for. While all households were asked, ahead of time, for permission to collect samples, they did not know exactly when swabbing would take place. This method was used to collect data on what bacterial contamination would be present at household sites during normal use and to reduce the impact of the cleaning strategies (of the different individuals) on the results.

### 2.4. Swabbing Methods 

Samples were collected using sterile swabs (BD CultureSwab™ BBL, Franklin Lakes, NJ, USA) inside of collection tubes containing a sponge with 1 mL liquid Amies solution (via Sinclair & Gerba, 2011 [15]). To maximize recovery, an additional 2 mL of sterile phosphate buffer solution (PBS) with 0.2% sodium thiosulfate was added to the collection tube, prior to swabbing. During swabbing, the swabs were aseptically removed from the transport tube and swabbed completely over the predefined surface area, before being returned to the collection tube for storage. The surface area swabbed varied based on the shape and size of surfaces. For small surfaces, including taps or handles on buckets used for anal cleansing, the entire surface was swabbed. For larger surfaces (walls and latrine slabs), a swabbing area was measured out, based on the expected contamination level on that surface and accounting for the surface shape (usually, 10 cm^2^ for wall surfaces and 5 cm^2^ for latrine slabs). Once collected, the swabs were transported on ice, to be stored at 4 °C, prior to processing (<30 h). A description of the types of surfaces swabbed are included in Table 1.

### 2.5. Lab Methods

Swabs were processed in the Environment and Public Health Organization (ENPHO) lab located in Kathmandu, Nepal, using membrane filtration methods. Samples were analyzed for concentrations of total coliforms and *Escherichia coli* (*E. coli*) which are used as indicators of fecal contamination. Swabs were vortexed in the Amies/PBS/sodium thiosulfate solution for 30 s to increase the removal of bacteria from the swab tip into the surrounding solution. The solution was then emptied into a sterile test tube and the swab tip and sponge were squeezed to remove any additional liquid (typically, 2–2.5 mL of solution was recovered). Next, 1 mL of the sample liquid was filtered through a 47 mm, 0.45 micron mixed cellulose filter, using membrane filtration methods. The filter was placed onto a prepared petri dish (47 mm) with Brilliance™ Total Coliforms/*E. coli* Selective Agar (Oxoid Microbiology Products, Cheshire, United Kingdom). All samples were processed in duplicates. For samples taken from surfaces with visibly higher levels of fecal contamination, serial dilutions (up to three dilutions) were done and were also processed in duplicates. Samples were then incubated for 24 h at 35 °C and total coliform and *E. coli* colonies were enumerated.

### 2.6. Statistical Methods 

Data were compared across the replicate latrine cabins, within each site-type, using the Kruskal-Wallis tests and between each site-type, or cleaning condition, using the Wilcoxon Signed Rank tests. All analyses were done using R version 3.4.1.

## 3. Results

A total of 96 swab samples were taken from the community toilet sites in Kathmandu, Nepal, on 5–7 March 2018, half of which were collected during “clean” and half of which were collected during “dirty” conditions. These include 54 samples from site 1 and 42 samples from site 2. An additional 23 swab samples were collected from the household sites in Kathmandu on 4 March 2018 and 24 swab samples were taken from the toilets located at the Temple University’s main campus as a method of comparison, in April 2018. Two money samples were collected from a community latrine operator to explore the possibility of money as a potential source of bacterial contamination.

Total coliform (TC) and *E. coli* (EC) concentrations recovered from the latrine surfaces are summarized in Table 2. Importantly, surfaces with the highest bacterial concentrations varied, depending on whether samples were collected from a community or household site. For example, the latrine slab (or toilet seat (TS)) had the highest median bacterial concentrations (median TC = 214/cm^2^ and median EC = 56/cm^2^) at the community sites (all of which were pit latrines), while the tap, spray handle, or bucket used for anal cleansing had the highest median bacterial contamination at the household sites (median TC = 1/cm^2^ and median EC = 0.4/cm^2^). In addition, very low bacterial concentrations were recovered from the university control site, with only small numbers of TC recovered from the door handle and floors.

Data from the two community toilet sites were compared using the Kruskal-Wallis tests to determine whether the latrine cabins were statistically similar enough to be used as replicates that could be analyzed together. In addition, data collected from the five household sites were also compared using the Kruskal-Wallis tests, to determine if there were statistically significant differences across sites. All Kruskal-Wallis tests found that each replicate household site and each replicate community latrine were not significantly different from each other (community sites included only replicate cabins, not handwashing stations) (Kruskal-Wallis results for household TC *χ*^2^(4) = 7.82, *p* = 0.10; for household EC *χ*^2^(4) = 7.83, *p* = 0.10; for community TC clean samples *χ*^2^(10) = 4.49, *p* = 0.922; for community EC clean *χ*^2^(10) = 5.14, *p* = 0.882; for community TC dirty *χ*^2^(10) = 6.89, *p* = 0.735; for community EC dirty *χ*^2^(10) = 6.19, *p* = 0.799). For this reason, data from all household and all community latrine sites were aggregated and analyzed together. In addition, because very few samples that were taken at the university control site had TC and EC concentrations above the method detection limit (1 cfu per surface area swabbed), these control sites were not statistically compared to the study sites in Kathmandu.

**Figure 1:**
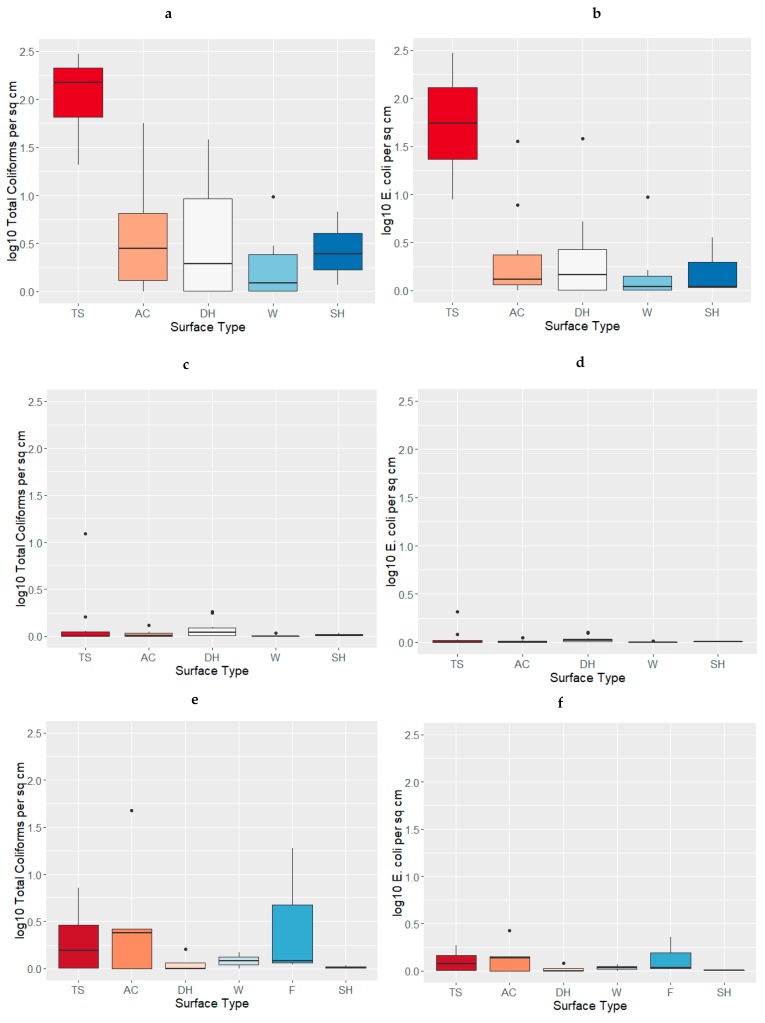
Bacterial Concentrations by Surface Type for Household and Community Latrines (boxplots represent median, 5% and 95% quartiles, range, and potential outliers). (**a**) Total Coliforms by Surface Type for Community Latrines under “Dirty” Conditions; (**b**) *Escherichia coli* (*E. coli*) by Surface Type for Community Latrines under “Dirty” Conditions; (**c**) Total Coliforms by Surface Type Community Sites under “Clean” Conditions; (**d**) *E. coli* by Surface Type Community Sites under “Clean” Conditions; (**e**) Total Coliforms by Surface Type Household Sites; (**f**) *E. coli* by Surface Type Household Sites.

Boxplots displaying the distribution of TC and EC concentrations collected at community (clean and dirty) and household toilet sites in Kathmandu, Nepal are displayed in Figure 1a–f. In addition, due to the small sample size and skewed data distributions, Wilcoxon-Mann-Whitney rank sum tests were done to test the significant differences between the bacterial concentrations, quantified on the community and household latrine surfaces. Results of the Wilcoxon-Mann-Whitney rank sum tests are displayed in Table 3, Table 4 and Table 5 below.

Table 3 displays the TC and EC concentrations recovered from the latrine surfaces in the community latrines, during both clean and dirty conditions. In all cases, the median bacterial concentration, per square centimeter, was higher before cleaning rather than after cleaning, however, this difference was only statistically significant for the toilet seat/latrine slab (TS, *p* < 0.01 for both TC and EC), tap/handle/bucket used for anal cleansing (AC, *p* = 0.01 for both TC and EC), and the wall (W, *p* = 0.01 for TC and *p* = 0.02 for EC). Results from this analysis suggest that cleaning practices were successful in significantly reducing bacterial contamination on latrine surfaces, at the community latrine sites.

Next, data comparing the bacterial concentrations recovered from household and both dirty and clean community latrine surfaces are summarized in Table 4 and Table 5 respectively. In Table 4, the only surface that was statistically different between dirty community latrine and household latrine surfaces was the toilet seat/latrine slab (*p* < 0.01), while some moderately significant differences (*p* = 0.10) were observed when comparing the EC concentrations on the door handles (DH), and both TC and EC concentrations on the sink handles used for handwashing (SH). In addition, as can be seen in Table 5, there were no statistically significant differences observed between the bacterial concentrations recovered at the household latrine and clean community latrine surfaces. As no organisms were recovered from the TS, F, W, DH, or SH, at any sample taken at the university control site, these data have not been included in Table 4 and Table 5.

## 4. Discussion

Results from this study found almost no difference in bacterial contamination on the latrine surfaces in household and community latrines in Kathmandu, Nepal. While a significant difference was observed when comparing the bacterial contamination on toilet seats/latrine slabs between community and household latrines, this type of surface might be less likely to play a role in hand-to-mouth contact (as compared to door handles, or other surface types) and for this reason, are also less likely to contribute to pathogen transmission via the fecal-oral route. Further, while all community toilet sites were pit latrine toilets, three out of the five household sites had cistern-flush style toilets. For this reason, it is likely that this difference was mainly due to the toilet construction rather than the maintenance or overall cleanliness of the latrine itself. Results identified the toilet seat/latrine slab, tap/handle/bucket used for anal cleansing, and door handles to the latrine cabins as the surfaces with the highest bacterial concentrations, in the community latrines. In the household latrines with flush toilets, the flush handle also appeared to have higher bacterial concentrations than the other surfaces. These results may have important implications for advising future cleaning protocols at community toilet sites and support the utility of community toilets in areas where household or private sanitation facilities are less practical due to space requirements, which is often the case in dense urban environments and informal settlements [8,16].

Cleaning practices at these sites are likely to play an important role in the results observed. At the community toilet sites in Kathmandu, cleaning occurred once a day at the end of the day. Cleaning practices involved rinsing the slabs with a bucket of water and wiping smaller surfaces with a clean rag with soap. No cleaning observations were made for the household sites. While it appeared that these cleaning strategies were effective for these community sites, there is currently no clear guidelines around the best practices for community latrine cleaning. Further, other factors such as seasonal variability (often due to flooding), water availability, construction materials, availability of cleaning materials, location, motivation, etc. were all thought to impact the cleaning practices at shared or community latrine sites [17,18,19]. As latrine cleanliness has been identified as a key issue in determining the acceptability and use of community toilet facilities (for both men and women) [20], it is important for future research to identify the best cleaning practices, as well as to identify the potential barriers to effective cleaning practices, at these sites.

While findings from this paper support previous research that suggests that shared/community sanitation facilities may not necessarily be less clean than private or household facilities [9], it is clear that there may be other important factors that contribute to pathogen exposure at these sites. For example, this study only sampled bacteria from latrine surfaces and did not look at direct exposure to users. In fact, certain factors, such as water and soap availability for handwashing, as well as social desirability around handwashing were not taken into account in the analysis, although they play a large role in disease transmission [21,22,23]. Previous research that measured bacterial transfer from surfaces to hands found that rates of transfer depended on both the surface type (with transfer the highest on hard, non-porous surfaces), as well as the organism [24]. This study also did not examine other transmission routes (e.g., airborne transmission and transmission via flies) that may also contribute to pathogen exposure. For this reason, latrine construction materials, design, local bacterial/pathogen communities, and unexplored transmission routes might also impact the direct disease risk to latrine users [9,19].

Other factors besides cleanliness and direct disease transmission could also impact the benefit that community toilets have on increasing access to improved sanitation facilities. These factors may include distance, waiting time, latrine maintenance, safety, privacy, and gender equity, which can all impact latrine usage [8,9,25,26,27]. Further, in this sample, both community latrine sites were pay-per-use which might also present a barrier for use, especially for low-income families. However, in a recent survey of women in Kathmandu, over half (55%) reported that they would not use the toilets more often if they were free-of-charge, suggesting that other factors such as cleanliness might play a larger role in latrine usage [26]. In addition, while exposure to bacterial contamination for latrine users might not differ across shared or household sites, waste management strategies that impact the surrounding water quality (local surface and drinking water) might be very different. In fact, waste disposal and treatment in community sanitation infrastructure might be better regulated and managed via community initiatives and local policy, while individual households may not have the capacity to properly treat and dispose of their waste.

This study was limited by a small sample size, with sampling conducted over a short time-period. For this reason, this study did not capture conditions that may vary across seasons or across geographical areas. To quickly conduct analyses on samples, this study utilized total coliforms and *E. coli* as indicator organisms, which are typically used to measure the presence of fecal contamination and potentially assess the presence of pathogens. However, due to different survivability and persistence in the environment, as well as different prevalence in fecal contamination, the presence of these bacteria do not necessarily correspond to the presence of pathogens [28]. Further, the use of non-flocked swabs might have also impacted the recovery of these organisms, as is suggested by the lack of bacterial contamination detected at the United States site [29]. However, it is unclear how the use of these particular swabs may have impacted recovery, as some research has found no difference between flocked and non-flocked swabs in other contexts [30,31]. In addition, similar methods have been used in previous research measuring bacterial contamination on inanimate surfaces [15]. For this reason, future research that looks specifically at pathogens rather than bacterial contamination, as well as research that examines direct exposure rather than only analyzing surface concentrations, is needed to better understand and compare cleanliness and disease risk between community and household latrine users.

## 5. Conclusions

While bacterial contamination on latrine surfaces might be highly dependent on local cleaning strategies and environmental and behavioral factors, our results suggest that well-maintained community latrines might be as clean as household latrines. For this reason, these results support the use of shared sanitation infrastructure in areas with a high need, including growing urban centers in low-income countries. These results might have important implications for planning future sanitation interventions and for guiding cleaning protocols for community latrines sites.

## Figures and Tables

**Table 1:** Description of surfaces swabbed.

Surface ID	Household Latrines			Community Latrines			
	Surface Type	Average Area Swabbed	*N*	Surface Type	Average Area Swabbed	*N* (Dirty)	*N* (Clean)
**TS**	Toilet seat for cistern flush toilets or latrine slab for pit latrines	76 cm^2^	5	Latrine slab	55 cm^2^	16	11
**AC**	Spray handle/bucket for anal cleansing	23 cm^2^	5	Tap used for anal cleansing	140 cm^2^	16	11
**DH**	Door Handle	82 cm^2^	5	Door Handle	30 cm^2^	16	11
**W**	Wall (Pit latrines only)	100 cm^2^	2	Wall	100 cm^2^	16	11
**SH**	Sink handle at handwashing station	424 cm^2^	3	Sink handle at handwashing station	218 cm^2^	6	3
**F**	Flush (cistern flush toilets only)	10 cm^2^	3	Flush (United States control site only)	28 cm^2^	6	1
**Fl**				Floor (United States control site only)	100 cm^2^	5	
**M**				Money (collected from latrine operator at community site 1) *	45 cm^2^	2	

* Two exploratory money samples were swabbed, to see if money could be a potential transmission route of concern that is associated with shared sanitation.

**Table 2:** Bacterial concentrations on latrine surfaces at community and household sites.

Site-Types	Surface Type	Sample *N*	Total Coliforms Median cfu/cm^2^	Total Coliforms Range	*E. coli* Median cfu/cm^2^	*E. coli* Range
Community-Dirty	TS	10	214	(20–1060)	56	(8–295)
	AC	10	2	(0–56)	0.3	(0–34)
	DH	10	1	(0–37)	0.5	(0–37)
	W	10	0.2	(0–9)	0.1	(0–8)
	SH	3	1	(0.2–6)	0.1	(0.07–3)
Community–Clean	TS	10	0.03	(0–11)	0	(0–1)
	AC	10	0.02	(0–0.3)	0.01	(0–0.1)
	DH	10	0.1	(0–0.8)	0.04	(0–0.3)
	W	10	0	(0–0.01)	0	(0–0.03)
	SH	3	0.02	(0.02–0.1)	0.01	(0–0.05)
Households	TS	5	0.5	(0–6)	0.2	(0–0.9)
	AC	5	1	(0–47)	0.4	(0–23)
	DH	5	0	(0–0.6)	0	(0–0.2)
	W	2	0.2	(0–0.5)	0.1	(0–0.2)
	F	3	0.2	(0.1–18)	0.08	(0.04–1)
	SH	3	0.03	(0–0.7)	0.01	(0–0.03)
Control Site (USA)	TS	5	0	NA	0	NA
	DH	5	0	(0–0.01)	0	NA
	F	5	0	NA	0	NA
	Fl	5	0	(0–0.1)	0	NA
	SH	3	0	NA	0	NA
Money *	M	2	0.2	(0–0.3)	0.03	(0–0.06)

* Two exploratory money samples were swabbed, to see if money could be a potential transmission route of concern that is associated with shared sanitation. *E. coli.:* *Escherichia coli.*

**Table 3:** Results of the Wilcoxon Signed Rank Test comparing the bacterial contamination of the community latrine surfaces, during the clean and dirty conditions (bolded if significant).

Surface Type	Total Coliforms			*E. coli*		
	Median (cfu/cm^2^) Before Cleaning	Median (cfu/cm^2^) After Cleaning	*p*-Value	Median (cfu/cm^2^) Before Cleaning	Median (cfu/cm^2^) After Cleaning	*p*-Value
TS	214.77	0	<0.01	56.25	0.003	<0.01
AC	1.90	0.02	0.01	0.30	0.01	0.01
DH	0.99	0.10	0.06	0.47	0.04	0.06
W	0.24	0	0.01	0.1	0	0.02
SH	1.46	0.24	0.25	0.09	0.01	0.25

**Table 4:** Results of the Wilcoxon Signed Rank Test comparing the bacterial contamination of the community latrine surfaces, during dirty conditions, to the household latrine surfaces (bolded if significant).

Surface Type	Total Coliforms			*E. coli*		
	Household Median (cfu/cm^2^)	Community Median (cfu/cm^2^) Before Cleaning	*p*-Value	Household Median (cfu/cm^2^)	Community Median (cfu/cm^2^) Before Cleaning	*p*-Value
**TS**	**0.55**	**214.77**	**<0.01**	**0.19**	**56.25**	**<0.01**
AC	1.39	1.90	0.67	0.38	0.30	0.67
DH	0	0.99	0.16	0	0.47	0.10
W	0.24	0.24	0.59	0.08	0.10	0.66
F	0.08	NA	NA	0.08	NA	NA
SH	0.03	1.46	0.10	0.01	0.09	0.10

**Table 5:** Results of the Wilcoxon Signed Rank Test comparing the bacterial contamination of community latrine surfaces, during clean conditions, to the household latrine surfaces (bolded if significant).

Surface Type	Total Coliforms			*E. coli*		
	Household Median (cfu/cm^2^)	Community Median (cfu/cm^2^) After Cleaning	*p*-Value	Household Median (cfu/cm^2^)	Community Median (cfu/cm^2^) After Cleaning	*p*-Value
TS	0.58	0	0.22	0.19	0.004	0.28
AC	1.39	0.02	0.50	0.38	0.01	0.50
DH	0	0.10	0.45	0	0.04	0.45
W	0.24	0	0.19	0.08	0	0.19
F	0.29	NA	NA	0.08	NA	NA
SH	0.03	0.02	1	0.01	0.01	1

## References

[1] World Health Organization/UNICEF (2017). Progress on Drinking Water, Sanitation and Hygiene: 2017 Update and SDG Baselines.

[2] Lim S.S., Vos T., Flaxman A.D., Danaei G., Shibuya K., Adair-Rohani H., Amann M., Anderson H.R., Andrews K.G., Aryee M. (2012). A comparative risk assessment of burden of disease and injury attributable to 67 risk factors and risk factor clusters in 21 regions, 1990–2010: A systematic analysis for the Global Burden of Disease Study 2010. Lancet Lond. Engl..

[3] Prüss-Üstün A., Bos R., Gore F., Bartram J. (2008). Safer Water, Better Health: Costs, Benefits and Sustainability of Interventions to Protect and Promote Health.

[4] Flores G.E., Bates S.T., Knights D., Lauber C.L., Stombaugh J., Knight R., Fierer N. (2011). Microbial biogeography of public restroom surfaces. PLoS ONE.

[5] Montgomery M.A., Elimelech M. (2007). Water and sanitation in developing countries: Including health in the equation. Environ. Sci. Technol..

[6] WHO/UNICEF (2015). Progress on Sanitation and Drinking Water—2015 update and MDG assessment.

[7] (2014). Progress on Drinking Water and Sanitation: 2014 Update.

[8] Heijnen M., Cumming O., Peletz R., Chan G.K.-S., Brown J., Baker K., Clasen T. (2014). Shared Sanitation versus Individual Household Latrines: A systematic review of health outcomes. PLoS ONE.

[9] Exley J.L.R., Liseka B., Cumming O., Ensink J.H.J. (2015). The sanitation ladder, what constitutes an improved form of sanitation?. Environ. Sci. Technol..

[10] Least Developed Country Category: Nepal Profile|Economic Analysis & Policy Division. https://www.un.org/development/desa/dpad/least-developed-country-category-nepal.html.

[11] (2014). World Urbanization Prospects: The 2014 Revision.

[12] Regmi L.K. (2015). An Overview of Population Growth Trends of Nepal. J. Inst. Sci. Technol..

[13] 2.6 Million Nepalis Still Lack Toilet—General—The Kathmandu Post. http://kathmandupost.ekantipur.com/news/2017-11-21/26-million-nepalis-still-lack-toilet.html.

[14] Nepal: Annual Household Survey 2015/16—Nepal. https://reliefweb.int/report/nepal/nepal-annual-household-survey-201516.

[15] Sinclair R.G., Gerba C.P. (2011). Microbial contamination in kitchens and bathrooms of rural Cambodian village households. Lett. Appl. Microbiol..

[16] Progress on Drinking Water and Sanitation: 2012 Update|UNICEF Publications|UNICEF. https://www.unicef.org/publications/index_69025.html.

[17] Kwiringira J., Atekyereza P., Niwagaba C., Kabumbuli R., Rwabukwali C., Kulabako R., Günther I. (2016). Seasonal variations and shared latrine cleaning practices in the slums of Kampala city, Uganda. BMC Public Health.

[18] Tumwebaze I.K., Mosler H.-J. (2014). Shared toilet users’ collective cleaning and determinant factors in Kampala slums, Uganda. BMC Public Health.

[19] Sonego I.L., Mosler H.-J. (2014). Why are some latrines cleaner than others? Determining the factors of habitual cleaning behaviour and latrine cleanliness in rural Burundi. J. Water Sanit. Hyg. Dev..

[20] Kwiringira J., Atekyereza P., Niwagaba C., Günther I. (2014). Gender variations in access, choice to use and cleaning of shared latrines; experiences from Kampala Slums, Uganda. BMC Public Health.

[21] Biran A., Schmidt W.-P., Wright R., Jones T., Seshadri M., Isaac P., Nathan N.A., Hall P., McKenna J., Granger S. (2009). The effect of a soap promotion and hygiene education campaign on handwashing behaviour in rural India: A cluster randomised trial. Trop. Med. Int. Health.

[22] Fewtrell L., Kaufmann R.B., Kay D., Enanoria W., Haller L., Colford J.M. (2005). Water, sanitation, and hygiene interventions to reduce diarrhoea in less developed countries: A systematic review and meta-analysis. Lancet Infect. Dis..

[23] Briscoe C., Aboud F. (2012). Behaviour change communication targeting four health behaviours in developing countries: A review of change techniques. Soc. Sci. Med..

[24] Rusin P., Maxwell S., Gerba C. (2002). Comparative surface-to-hand and fingertip-to-mouth transfer efficiency of gram-positive bacteria, gram-negative bacteria, and phage. J. Appl. Microbiol..

[25] Tiimub B.M., Forson M.A., Obiri-Danso K., Rahaman I.A. (2009). Pointed gaps in the provision, quality, patronage and management of toilet facilities in Bawku East District. Water, Sanitation and Hygiene: Sustainable Development and Multisectoral Approaches, Proceedings of the 34th WEDC International Conference, United Nations Conference Centre, Addis Ababa, Ethiopia, 18–22 May 2009.

[26] Monsell L., Dullaghan N., Krastev S., Pilat D. (2018). Applying Behavior Change to Promote Gender—Symmetrical Public Toilet Usage in Nepal.

[27] Patterns and Determinants of Communal Latrine Usage in Urban Poverty Pockets in Bhopal, India. https://www.ncbi.nlm.nih.gov/pubmed/21414114.

[28] Savichtcheva O., Okabe S. (2006). Alternative indicators of fecal pollution: Relations with pathogens and conventional indicators, current methodologies for direct pathogen monitoring and future application perspectives. Water Res..

[29] Moore G., Griffith C. (2007). Problems associated with traditional hygiene swabbing: The need for in-house standardization. J. Appl. Microbiol..

[30] Wigger C., Morris P.S., Stevens M., Smith-Vaughan H., Hare K., Beissbarth J., Leach A.J. (2019). A comparison of flocked nylon swabs and non-flocked rayon swabs for detection of respiratory bacteria in nasopharyngeal carriage in Australian Indigenous children. J. Microbiol. Methods.

[31] Chavda K.D., Satlin M.J., Chen L., Manca C., Jenkins S.G., Walsh T.J., Kreiswirth B.N. (2016). Evaluation of a Multiplex PCR Assay to Rapidly Detect Enterobacteriaceae with a Broad Range of β-Lactamases Directly from Perianal Swabs. Antimicrob. Agents Chemother..

